# Understanding household maternal and child health predictors: a periodic assessment across three states in Sudan

**DOI:** 10.3389/fpubh.2026.1697577

**Published:** 2026-10-14

**Authors:** Geoffrey Babughirana, Ali Salah, Habob Modawy, Ishag Adom, Ali Hado, Tonny Omondi, Daniel Kheri Kazungu, Yosh Kasilima

**Affiliations:** 1 Strategy, Program Quality and Evidence Department, World Vision Sudan, Port Sudan, Sudan; 2 Independent Researcher, Mogadishu, Somalia

**Keywords:** breastfeeding, conflict, dietary diversity, emergency nutrition, health services, maternal and child nutrition, Sudan

## Abstract

**Background:**

While Sudan's essential health care package historically aimed to support maternal and child health services, limited empirical evidence exists on patterns of service utilization, health-seeking behaviors, and maternal, infant, and young child nutrition practices under the current crisis conditions. This study set out to examine maternal and child health service use, health-seeking behaviors, and nutrition practices in three Sudan states in 2021 through 2024.

**Methods:**

Annual repeated cross-sectional household surveys were conducted from 2021 to 2024 across three Sudanese states: Blue Nile, South Darfur, and East Darfur. Each year, approximately 906 households were sampled using probability-proportional-to-size (PPS) cluster sampling. Data collection occurred during the post-harvest season using a standardized questionnaire covering household demographics, infant and young child feeding (IYCF) practices, health service utilization (antenatal care, delivery, immunization), and key nutrition indicators. The tools were translated into local languages, pretested, and administered electronically using KoboCollect. Descriptive statistics and trend analyses were performed in SPSS version 25, with results presented as frequencies, means, and logistic regression analyses were 95% confidence interval was determined.

**Results:**

Although any ANC attendance remained high (87.3% on average), facility delivery was low (mean 23.1%) and home births predominated (mean 76.9%), suggesting sustained barriers to safe childbirth access. Nutritional risks widened: ever breastfeeding declined (91.8% to 82.7%), continued breastfeeding fell sharply (99.1% to 70.4%), and pre-lacteal feeding increased to 64.5% in 2024, patterns consistent with documented IYCF vulnerabilities during crises. Bivariate and multivariate logistic regression analyses were done where Female caregivers demonstrated higher odds of achieving minimum dietary diversity compared with male caregivers (OR ≈ 2.4, *p* < 0.001). Female caregivers had higher adjusted odds of practicing appropriate newborn care (aOR≈3.4, *p* < 0.001).

**Conclusion:**

The collapse of Sudan's health systems compounded by recurrent shocks has significantly eroded MIYCN and maternal health service uptake, with gains in 2022 largely reversed by 2024. These reductions in maternal new-born and child health survive means that Sudan might not be able to meet its targets as stipulated in every Woman, every child loba framework. Further Sudan might not be able to substantially to meet the Sustainable development goals 2 (Zero Hunger), 3(Good Health and wellbeing) and 5 (Gender Equality).

## Background

Sudan is currently grappling with one of the most critical health and nutrition emergencies, driven by protracted armed conflict, economic collapse, and repeated climate shocks (1, 2). These overlapping crises have deeply eroded health systems, disrupted food production and access, and triggered widespread displacement, placing millions at risk of malnutrition, disease, and preventable mortality (3, 4). As of 2024, over 30 million people, including 16 million children, require humanitarian aid, with 1.3 million children at risk of famine and 770,000 projected to suffer from severe acute malnutrition (5). Acute food insecurity has worsened dramatically, with the number of food-insecure individuals rising from 9.6 million in 2020 to 25.6 million by mid-2024, standing for more than half of Sudan's population (1, 2). Conflict-affected regions such as Greater Darfur, Greater Kordofan, Khartoum, and Al Jazirah remain the hardest hit (6).

The health system has collapsed under the strain. Between 70%−80% of hospitals have ceased operations, and community-based health and nutrition services such as outpatient therapeutic feeding and community health worker (CHW) outreach have declined sharply (7). Antenatal care coverage fell from 60% in 2020 to 45% in 2024 in conflict-affected areas (8), while child immunization rates, which once reached 78%, plummeted to 55% due to health facility disruptions and insecurity (9). Despite resilience efforts such as localized “health mapping” and mobile outreach clinics service delivery remains sporadic and uneven, particularly in rural or displaced populations (10). There is a linkage between conflict, healthcare disruptions, and nutrition outcomes. Conflict drives malnutrition primarily by disrupting the disease environment, impairing food systems, and destroying healthcare infrastructure. This disruption leads to increased morbidity and mortality rates, particularly among women and children (11).

The Essential Health Care Package (EHCP) has historically served as Sudan's core strategy for delivering primary health services, including maternal, newborn, and child health (MNCH), nutrition, and psychosocial support (12). By early 2020, 85% of primary health centers were delivering a full EHCP suite (13). However, the onset of COVID-19 and escalating conflict from 2023 onwards reversed these gains. Supply chains were severed, health budgets slashed by hyperinflation, and CHW deployment shrank significantly, especially in insecure areas like Darfur and Kordofan (14).

In this context, the current study set out to examine healthcare service use, health-seeking behaviors, and nutrition practices within the fragile and volatile context of Sudan. It focuses on three states Blue Nile, South Darfur, and East Darfur where a four-year health and nutrition project was implemented. The study aims to generate empirical evidence on trends in service use, health-seeking behaviors, and nutrition practices among vulnerable populations. Given the erratic nature of humanitarian programming and service delivery in conflict settings, the findings will serve not only to check the status quo but also to inform future health and nutrition intervention design and policy advocacy. Past studies have failed to provide consistent repeat cross-sectional evidence in Sudan to guide policy makers on the trends in maternal new-born and child health on a conflict zone like Sudan and this study uniquely provides this opportunity.

## Methods

### Study area

The household surveys were conducted in three states in Sudan: Blue Nile, South Darfur, and East Darfur, which have been significantly affected by the ongoing conflict and humanitarian crises. The study aimed to capture a broad spectrum of data from various localities within each of these states to understand the health and nutrition dynamics at the grassroots level. In Blue Nile State, the survey covered the following localities: Kurmu,Tadamon, Bau, Alrosris, Damazine, and Giessan. These areas stand for a mix of rural and urban settings, offering insights into both isolated and more densely populated communities, which are crucial for understanding the regional variation in access to health services, food security, and overall wellbeing. In South Darfur, data were collected in Muhajiriya, Daora, and Mohgrua localities. These areas are characterized by their remote settings and exposure to the impacts of conflict, including displacement, loss of livelihood, and disruptions in healthcare access. The survey aimed to assess the effects of these factors on maternal and child health, malnutrition rates, and general health service delivery in these areas. Lastly, food insecurity and limited health infrastructure, making them key areas for understanding the broader health and nutrition challenges faced by vulnerable populations, particularly in the context of emergency settings (15).

### Study design

This study employed a Repeat cross-sectional survey design to assess household-level access to health and nutrition services across the selected regions of Sudan. This approach allowed for the collection of data at a single point in time, providing an effective means to evaluate the current state of health and nutrition among households and to find immediate barriers to service access and utilization. The survey was conducted during the post-harvest period, a critical time when food stocks (16) at the household level are typically low, and dependency on markets for food supplies increases. During this period, many households face heightened vulnerability, with a potential increase in food insecurity and malnutrition. Moreover, the post-harvest period often coincides with seasonal challenges such as flooding, which can disrupt transportation routes and access to both markets and health services. The timing of the survey, therefore, allowed for an assessment of how these seasonal dynamics impact the availability and utilization of health and nutrition services. The same design was utilized throughout the multi-year evaluation of the interventions designed from the baseline survey in 2021.

This design was preferred because it collects data from a new, independent sample of a population at multiple time points receiving the same intervention. Instead of tracking the same individuals, it provides successive snapshots of the overall group to measure aggregate-level changes over time. This therefore eliminates participant fatigue and prevents individuals from altering their behavior because they know they are being studied. It is also highly cost-effective and avoids attrition over long periods.

### Sample size determination

Sample size for this multi-year assessment was determined to estimate key maternal, infant, and young child nutrition (MIYCN) and health service indicators with adequate precision across conflict-affected states in Sudan. For each survey round conducted between 2021 and 2024 in Blue Nile, South Darfur, and East Darfur, a conservative prevalence estimate of 50% was applied for core indicators. This approach is commonly recommended in population-based surveys when prior prevalence estimates are unavailable or uncertain, as it yields the maximum sample size required to achieve the desired statistical (17).

To account for the clustered nature of village-level sampling, a design effect of 1.5 was initially applied in 2021. This value is consistent with recommendations from humanitarian and nutrition survey methodologies indicating that cluster sampling typically increases variance relative to simple random sampling due to intra-cluster correlation in health and nutrition (18). The design effect reflects expected intra-cluster similarity in behaviors and access to services within villages. An initial 10% non-response adjustment was included to account for potential refusals and incomplete interviews under challenging field conditions. These parameters generated a minimum annual sample target of approximately 634 households, distributed across 36 village clusters in the three states. In practice, field data collection exceeded this minimum requirement. Accordingly, 2021 had 793, 2022 had 1,150, 2023 had 500 and 2024 had 899 respondents. This increased statistical precision and enabled subgroup analyses by locality, household composition, and service exposure.

Beginning in 2022 and maintained through 2024, the sampling parameters were adjusted to reflect evolving field realities. Due to frequent population displacement, returnee movements, and increased heterogeneity between clusters, the non-response allowance was increased to 20%, and the design effect was revised to 2.0 to account for greater intra-cluster variability in conflict-affected settings. These adjustments were made to maintain statistical reliability under conditions of increased population mobility and cluster heterogeneity. Maintaining a consistent sampling framework across successive rounds allowed the study to generate a comparable dataset suitable for assessing trends in household access to health and nutrition services over the four-year period.

### Sampling criteria and sampling procedures

The household selection process followed a multi-stage, stratified random sampling approach to ensure that the selected households are representative. The first stage of the sampling strategy involved stratifying the sampling frame to ensure adequate representation across the geographic area. These stratification layers will help ensure that the evaluation sample reflects the geographic diversity, intervention scope, and beneficiary composition of the overall project population.

The second stage of the sampling process involved the selection of enumeration areas within each Cluster (Village) where project activities were implemented. The evaluation applied Probability Proportional to Size (PPS) sampling. Under this communities with a larger number of beneficiary households had a higher probability of being selected compared to smaller communities. The primary sampling frame was constructed using beneficiary databases and distribution records to generate sampling weights.

The third stage of the sampling process involved selecting individual households in the communities. This stage is critical to ensuring that the households interviewed are representative of the beneficiary population. Once the beneficiary list for a given community was finalized, the sampling interval was calculated to determine how households were systematically selected.

The next step will involve identifying the most appropriate respondent within that household. The primary respondent was ideally the household head, as this individual is generally responsible for decision-making, in situations where the household head is unavailable at the time of the visit, another adult household member aged 18 years or older was selected as the respondent, provided that the individual is sufficiently knowledgeable about the household's activities.

Despite careful planning, situations may arise where a selected household is unavailable during the survey visit, has relocated, or declines to participate in the evaluation. In such cases, a replacement procedure was applied to ensure that the required sample size is maintained without compromising the integrity of the sampling design.

### Tool development and pretest

Tools were initially drafted in English and subsequently translated into Arabic through a dual translation process to ensure linguistic and cultural accuracy. Pretesting was conducted in non-sampled villages within the same localities, allowing the team to refine ambiguous items and adjust the flow of questions based on respondent comprehension. Following these adjustments, the final tool was deployed using KoboCollect on tablets, with embedded checks for logic, range, and skip patterns to ensure data quality during collection (18).

The study tool was checked for validity and reliability to ensure it is well tested for accuracy and consistency. Validation was done through having two experts review every item in the tool to ensure all parts of the topics are covered. This meant the tool was able to measure what it should measure. Reliability was done where two different versions of the tool were made and given to the same person to review and the scores were compared. With the same scores this meant the tool gave the same stable results every time you use it under the same condition.

### Variables/measures for the study

The study assessed household characteristics, health service utilization, and maternal, infant, and young child nutrition practices. The primary outcomes were: (i) appropriate infant and young child feeding (IYCF) practices, measured through indicators including ever breastfeeding, continued breastfeeding, pre-lacteal feeding, early initiation of breastfeeding, use of colostrum, reasons for stopping breastfeeding, and age at introduction of complementary foods; (ii) child dietary diversity, assessed using a 24-h recall and classified according to minimum dietary diversity (consumption of ≥5 of 8 food groups including breastmilk) vs. low dietary diversity; (iii) maternal and newborn health service utilization, measured through place of delivery, type of delivery assistance, antenatal care attendance (at least one visit and ≥4 visits), and postnatal care attendance within six weeks postpartum; and (iv) immediate newborn care practices, including immediate drying, early breastfeeding, skin-to-skin contact, head covering, early bathing, and mother–infant separation. Secondary outcomes included newborn immunization at birth (BCG and oral polio vaccine) and cord care practices. These outcomes were analyzed in relation to key explanatory variables describing household and respondent characteristics, including locality, village, household size, respondent age, sex, education (literacy), occupation, marital status, and population displacement status (host, returnee, or internally displaced person).

### Data collection methods

Data collection was conducted by trained enumerators fluent in Arabic and familiar with the cultural contexts of the selected localities. A two-day training was held prior to deployment, covering tool content, consent protocols, gender-sensitive interviewing, and digital data entry procedures (19). Enumerators practiced through mock interviews and were supervised closely during initial field days. Field teams were assigned based on gender balance and local language fluency to ensure comfort and trust, especially when engaging pregnant and breastfeeding women and girls. Supervisors conducted routine spot checks, reviewed daily submissions for inconsistencies, and provided feedback for corrections in real-time. KoboCollect was used to capture data electronically, which reduced errors and facilitated rapid uploads to a centralized server. The deployment period spanned three weeks and included real-time coordination with community leaders to navigate accessibility issues in flood-prone or remote locations.

#### Inclusion and exclusion criteria

For a household participate to be included, there must have been a child below one year, the respondent should be of consent age, 18 years and above, have lived in the study area for at least 12 months, the household must have a pregnant or lactating woman and the child should have been born with in the study area.

Data inclusion into this study was that where a recall period of 90 days was in effect.

### Data analysis and interpretation

Following data cleaning in Excel and SPSS-v25, the dataset underwent a structured descriptive statistical analysis. Cleaning involved checking for duplicate entries, resolving missing values where possible, and validating skip patterns and logical consistency between variables (18). Once finalized, the dataset was subjected to frequency analysis to generate proportions and distributions for all key categorical variables. Means, standard deviations, minimum, and maximum values were calculated for continuous variables such as household size, age of respondents, and dietary diversity scores for both pregnant and lactating women and children under five. Data from the four survey rounds (2021–2024) were harmonized by variable names and pooled into a single dataset. Descriptive statistics summarized participant characteristics and key indicators. Bivariate logistic regression assessed associations between independent variables and each outcome.

Subgroup analyses were conducted to explore variations in key outcomes across selected population characteristics, including locality, population displacement status (host community, internally displaced persons, and returnees), household size categories, caregiver literacy status, and marital status. Multivariate logistic regression was utilized to estimate adjusted odds ratios (aOR) and 95% confidence intervals for predictors of appropriate infant feeding practices, dietary diversity, maternal and newborn health service utilization, and immediate newborn care practices. Multicollinearity was done to ascertain whether two or more independent variables were highly correlated with each other. This was to avoid confusion in the model, leading to unstable, unreliable regression coefficients and inflated standard errors. Temporal effects were modeled during analysis to capture change over time, including trends, seasonality, and sequential dependencies. Common methods used included time series analysis, recurrent neural networks, and fixed effects regression.

Missing data during analysis was handled by identifying why values are absent and choosing between deletion, imputation, or specialized algorithms. The primary strategy was removing incomplete records. Further Variables for regression models were selected by balancing model simplicity and predictive power. subject-matter theory, and statistical metrics *p*-values were therefore included to ensure meaningful predictors while removing redundant or irrelevant data.

### Ethical considerations and approval

The assessment was conducted as part of a health system evaluation under the auspices of the Federal and State Ministries of Health. Ethical approval was obtained through the Sudan National Health Research Ethics Committee (19) and registered as (***6–12–22***), which deemed it a non-interventional systems assessment. Permissions were also secured from each State Ministry of Health and local authorities. Enumerators read out a consent statement in Arabic explaining the purpose of the survey, the voluntary nature of participation, what data would be collected, and assurances of confidentiality. Only those who provided verbal consent proceeded with the interview. Respondents (and community leaders) were made aware that the survey was for evaluation purposes and that refusing or discontinuing would not affect any services they receive. Privacy and confidentiality were strictly maintained. Interviews were conducted in locations where respondents felt comfortable to speak freely, usually inside or just outside their homes, but out of earshot of neighbors. No names were recorded on survey forms; instead, anonymous ID codes were used. The data files were kept confidential and used only for the purposes of this project assessment. In presenting results, only aggregate information is reported, and no individual can be identified.

## Results

### Demographics and social factors

Majority of the respondents were female for all the years of the study. 65.1% and 68.9% for 2021 and 2024 respectively. Married status dominated across the different years making up the majority and ranging from 82.6% (2022) to 89.7% (2024) suggesting that the target population is mainly composed of married status. Widowed respondents are the second highest category overall; single individuals show moderate presence in the target population.

Literacy remains a big challenge with slightly over half (51.2%) respondents on average over the years unable to read and write. The lowest literacy rate was in 2022 (44.0%) and 2024 shows improvement in literacy with 53.7% respondents able to read and write. There was an increase in House wife as an occupation status among women additionally, self-employment peaked in 2023 at 29.7%.

Displacement status varied across the survey years, with host community members constituting the largest proportion of respondents overall, although their share declined from 52.6% in 2021 to 40.9% in 2024. Internally displaced persons (IDPs) represented a substantial portion of the sample, increasing from 27.2% in 2021 to 34.6% in 2023, before declining to 20.7% in 2024. The proportion of returnees increased markedly between 2021 and 2023, rising from 13.9% to 33.6%, and remained relatively high in 2024 (25.0%). Refugees consistently accounted for a small proportion of respondents across all years, while the “other” displacement category increased notably in 2024 (10.0%) compared to earlier survey rounds as shown in Table 1.

**Table 1:** Socio-demographic characteristics of respondents by year (2021–2024).

Characteristic	2021 (*n* = 793)	2022 (*n* = 1150)	2023 (*n* = 500)	2024 (*n* = 899)
Sex				
Female	516 (65.1)	792 (68.9)	349 (71.8)	658 (73.2)
Male	277 (34.9)	358 (31.1)	141 (28.2)	241 (26.8)
Marital status				
Married	671 (84.6)	950 (82.6)	422 (84.6)	806 (89.7)
Single	50 (6.3)	51 (4.4)	14 (2.8)	50 (5.6)
Widowed	51 (6.4)	103 (9.0)	42 (8.4)	28 (3.1)
Divorced	18 (2.3)	34 (3.0)	18 (3.6)	13 (1.4)
Separated	3 (0.4)	12 (1.0)	3 (0.6)	2 (0.2)
Education				
Literate	392 (49.4)	506 (44.0)	239 (48.2)	482 (53.7)
Illiterate	401 (50.6)	644 (56.0)	261 (51.8)	416 (46.3)
Occupation				
Farmer	417 (52.6)	593 (51.6)	133 (26.7)	161 (17.9)
Housewife	158 (19.9)	268 (23.3)	118 (23.6)	502 (55.8)
Self-employed	112 (14.1)	157 (13.7)	148 (29.7)	98 (10.9)
Civil servant	26 (3.3)	35 (3.0)	58 (11.6)	49 (5.5)
Other occupations	80 (10.1)	97 (8.4)	42 (8.4)	89 (9.9)
Displacement status				
Host community	417 (52.6)	522 (45.4)	158 (31.6)	367 (40.9)
Internally displaced	216 (27.2)	271 (23.6)	173 (34.6)	186 (20.7)
Returnees	110 (13.9)	332 (28.9)	168 (33.6)	224 (25.0)
Other status	50 (6.3)	25 (2.2)	1 (0.2)	120 (13.4)
Household size category				
1–2 members	15 (1.9)	44 (3.8)	7 (1.4)	11 (1.2)
3–4 members	116 (14.6)	188 (16.3)	59 (11.7)	156 (17.3)
5–6 members	198 (25.0)	296 (25.7)	109 (21.7)	252 (28.0)
7–8 members	209 (26.3)	315 (27.4)	121 (24.2)	207 (23.0)
9–10 members	163 (20.5)	193 (16.8)	107 (21.4)	127 (14.1)
11–15 members	80 (10.1)	95 (8.3)	80 (16.0)	116 (12.9)
>15 members	12 (1.6)	19 (1.7)	18 (3.6)	30 (3.4)

The demographic variables have been summarized in Table 1.

### Patterns of service utilization

Maternal and newborn health service use was mixed across 2021–2024. Home deliveries remained dominant overall, averaging 76.9%, although health facility births improved markedly in 2023 before dropping again in 2024. Midwives were the main delivery support and their involvement increased over time, while non-skilled delivery support declined notably, which suggests some improvement in skilled attendance during childbirth. Antenatal care attendance was consistently high, with 87.3% of women attending at least one visit on average. However, continuity of care weakened over time. The proportion of pregnant women completing four or more ANC visits fell from about three-quarters in 2021–2022 to 51.7% in 2023 and only partly recovered to 57.2% in 2024.Simulteneously, single-visit attendance increased from 0% in 2021 to 12.2% in 2024 detailed in Table 2. Overall, this suggests strong initial contact with maternal health services, with declining adherence to the recommended ANC schedule.

**Table 2:** Maternal health service utilization, place of delivery and antenatal care (ANC) attendance by year (2021–2024).

Indicators	Category (%)	2021 (%)	2022 (%)	2023 (%)	2024 (%)
Birth place	Home	86.4	86.2	56.6	78.5
	Health facility	13.6	13.8	43.4	21.5
ANC attendance	Yes	87.0	88.3	85.1	88.7
	No	13.0	11.7	14.9	11.3
ANC times	One	0.0	0.9	4.2	12.2
	Two	9.7	4.6	7.7	13.1
	Three	17.7	19.9	36.4	17.5
	4 times and above	72.6	74.6	51.7	57.2

### Health-seeking behaviors

PNC attendance among mothers varied across the years. In 2021, 54.7% of mothers reported receiving postnatal care, which slightly decreased to 51.0% in 2022. There was a marked improvement in 2023, reaching a high of 68.6%, before declining again in 2024 to 49.9%, indicating inconsistent utilization of postnatal care services over time. Immediate newborn care practices showed varied changes between 2023 and 2024. Immediate bathing of newborns remained uncommon, increasing slightly from 3.2% to 5.2%, while head covering after birth increased from 0.4% to 6.1%. Placement of newborns in an incubator remained rare and declined from 1.4% to 0.6%. Early initiation of breastfeeding increased substantially from 0.4% in 2023 to 26.1% in 2024, representing the largest improvement among newborn care practices. Skin-to-skin contact also increased modestly from 0.7% to 6.1%, while the proportion of babies taken away from their mothers immediately after birth remained very low, rising slightly from 0.7% to 0.9%. Conversely, the proportion of newborns who were thoroughly dried immediately after birth declined markedly from 80.0% in 2023 to 50.5% in 2024. Newborn immunization coverage remained relatively high and improved slightly, with BCG vaccination increasing from 82.0% to 85.7% and Polio 0 vaccination from 72.9% to 83.4%. Cord care practices showed prominent shifts, with the use of medicinal substances declining from 31.6% to 13.2%, and the proportion of mothers applying nothing on the cord decreasing from 40.4% to 24.0%. Application of unspecified substances also declined slightly (26.7% to 21.3%), while the use of traditional herbs increased sharply from 1.4% in 2023 to 41.4% in 2024, representing the most substantial change in cord care practices over the period represented in Table 3.

**Table 3:** Newborn care practices and immediate postnatal interventions by year (2021–2024).

Practices (%)		2021	2022	2023	2024
PNC attendance	PNC visit	54.7	51.0	68.6	49.9
Practices in the labor suite/at home immediately baby was born	Bathed immediately	-	-	3.2	5.2
	Head covered with a Cap	-	-	0.4	6.1
	Immediately placed in an incubator	-	-	1.4	0.6
	Immediately put on the Breast	-	-	0.4	26.1
	Injections were given	-	-	13.2	4.6
	Placed skin to skin	-	-	0.7	6.1
	Taken away from the mother	-	-	0.7	0.9
	Thoroughly dried immediately	-	-	80.0	50.5
Baby immunized for BCG	Yes	-	-	82.0	85.7
Baby Immunized for Polio 0	Yes	-	-	72.9	83.4
What did the mother apply on the Cord before it broke off	Medicine (sprit, iodine)	-	-	31.6	13.2
	Nothing	-	-	40.4	24.0
	Others specify	-	-	26.7	21.3
	Traditional herbs	-	-	1.4%	14.4%

### Maternal, infant, and young child nutrition Practices

Comparisons are made across years where data is available, with particular emphasis on the most recent years of data collection (2023 and 2024) for practices. The proportion of children ever breastfed decreased from 91.8% in 2021 to 82.7% in 2024, while those still breastfeeding dropped even more sharply, from 99.1% to 70.4%. Breastfeeding frequency worsened between 2023 and 2024, with the share of children breastfed fewer than 8 times per day increasing sharply from 32.3% to 66.7%, while more frequent breastfeeding declined. At the same time, pre-lacteal feeding increased substantially, from 23.2% in 2021 to 64.5% in 2024, peaking at 67.8% in 2023, suggesting growing early introduction of non-breastmilk feeds.

Early breastfeeding practices showed mixed trends. Initiation within 1 h improved from 70.6% in 2021 to 84.0% in 2023, but this gain was lost in 2024 when it fell back to 70.4%. Colostrum feeding remained consistently high overall, averaging 89.6%, which stands out as one of the stronger practices across the period. The pattern of breastfeeding intentions and cessation also changed markedly. In 2024, 87% of mothers planned to breastfeed only 0–6 months, a sharp rise from 13.9% in 2023, pointing to a major shift toward shorter intended breastfeeding duration. Among reasons for stopping breastfeeding, “child had reached weaning age” remained common, but the sharp rise in unspecified “other” reasons from 18.6% in 2023 to 54.5% in 2024 is particularly notable and may indicate changing or poorly captured drivers of cessation. Bottle feeding remained a minority practice but increased from 8.7% in 2023 to 11.0% in 2024, reinforcing the broader pattern of movement away from optimal breastfeeding practices detailed in Table 4.

**Table 4:** Trends in infant and young child feeding (IYCF) practices by indicator and year (2021–2024), including averages for key indicators.

Indicator	Category	2021 (%)	2022 (%)	2023 (%)	2024 (%)	Average
Ever breastfed	Yes	91.8	91.9	85.4	82.7	88.0
Still breastfeeding	Yes	99.1	97.2	82.5	70.4	87.3
Frequency of breastfeeding	Low (< 8/day)			32.3	66.7	49.5
	Adequate (8-12/day)			44.5	27.4	36.0
	High (>12/day)			23.2	6.0	14.6
Planned breastfeeding duration	0–6 months			13.9	87	50.4
	7–12 months			6.3	7	6.65
	13–24 months			75.3	66	70.65
	>24 months			4.4	11	7.7
Bottle use the day before	Don't know			1.7	2.6	2.2
	No			89.7	86.4	88.1
	Yes			8.7	11.0	9.9
Solid/Semi-solid feeding the day before	None			11.4	2.1	6.8
	1–2 times			31.3	37.4	34.4
	34 times			50.7	52.4	51.6
	5+ times			6.5	8.0	7.3
Reason for cessation of breastfeeding	Became pregnant			17.1	9.0	13.1
	Mother ill/weak			10.0	0.5	5.3
	Mother working			7.1	0.5	3.8
	Not enough milk			2.9	1.6	5.3
	Other			18.6	54.5	36.6
	Started using contraception			4.3	2.1	3.2
	Weaning age/age to stop			40.0	31.7	35.9
Age at first introduction of semi-/solid foods	Don't know			8.9	4.7	6.8
	Less than 6 Months			54.7	36.1	45.4
	More than 6 months			35.4	58.1	46.8
Dietary diversity	Low dietary diversity	**-**	77.6	29.9	33.8	47.1
	High dietary diversity	**-**	22.4	70.1	66.3	52.9

#### Age at first introduction of solid foods

Early introduction of solid foods was high in 2023 (54.7) but declined to 36.1 in 2024, though the average of 45.4 still signals poor adherence to exclusive breastfeeding. Between 2021 and 2024, most caregivers reported not introducing feeds to infants aged 0–6 months, with 76.8% in 2021 and 56.8% in 2024 indicating “No.” However, there was a gradual rise in those who reported introducing feeds during this period, increasing from 23.2% in 2021 to 40.9% in 2024. A small proportion of respondents were unsure, with 3.8% in 2023 and 2.3% in 2024 stating “Don't know,” while no uncertainty was reported in 2021 and 2022. On average, 6.8% of caregivers did not know when solid or semi-solid foods were first introduced, 45.4% introduced them before six months, and 46.8% after six months.

#### Minimum dietary diversity

Dietary diversity improved over time, with the proportion of children meeting minimum dietary diversity consuming at least five food groups including breastmilk improving from 22.4 in 2021 to a peak of 70.1 in 2022, then slightly declining to 66.2 in 2023 and 52.9 in 2024. On average, over half of the children (52.9%) had high dietary diversity, reflecting moderate progress in complementary feeding. However, the drop in 2024 suggests setbacks likely linked to conflict or food insecurity. Low dietary diversity declined from 77.6 in 2021 to 29.9 in 2022 but increased again to 47.1 in 2024, highlighting the need for consistent support to sustain dietary gains.

### Bivariate and multivariate logistic regression

Bivariate and multivariate logistic regression analyses were conducted using pooled data from four survey years (2021–2024). At bivariate associations, for appropriate IYCF practices, marital status showed a significant association with the outcome. Married caregivers were more likely to report appropriate feeding practices compared to unmarried caregivers (OR ≈ 2.1, *p* < 0.01). Caregiver sex and household size were not significantly associated with IYCF practices. Differences across survey years were modest and not statistically significant after accounting for variability in the pooled sample.

For adequate dietary diversity, several predictors were associated with the outcome in the bivariate analysis. Female caregivers demonstrated higher odds of achieving minimum dietary diversity compared with male caregivers (OR ≈ 2.4, *p* < 0.001). Married caregivers were also more likely to achieve adequate dietary diversity (OR ≈ 2.7, *p* < 0.001). Household size showed an inverse association, indicating that larger households were less likely to achieve minimum dietary diversity (OR ≈ 0.82, *p* < 0.01). Temporal variation was observed across survey years, with higher odds of adequate dietary diversity in 2023 and 2024 relative to the 2021 baseline.

For MNH service uptake, marital status was the only predictor significantly associated with completion of the MNH service package in the unadjusted model (OR ≈ 3.0, *p* < 0.01). Caregiver sex and household size were not significantly associated with service uptake. Year-to-year differences were observed but did not reach statistical significance.

For immediate newborn care practices, caregiver sex, marital status, and household size were significantly associated with the outcome. Female caregivers were more likely to report appropriate newborn care practices compared with male caregivers (OR≈2.8, *p* < 0.001). Married caregivers also showed higher odds of appropriate newborn care practices (OR≈2.0, *p* < 0.001). Increasing household size was associated with reduced odds of appropriate newborn care (OR≈0.78, *p* < 0.001). Survey year comparisons suggested modest variation, with slightly lower odds observed in later survey years relative to the 2021 baseline as shown in Table 5.

**Table 5:** Bivariate regression analysis unadjusted odds ratio between predictors and outcomes.

Predictor	Category	Appropriate IYCF Practices OR (95% CI)	*p*-value	Adequate Dietary Diversity OR (95% CI)	*p*-value	MNH Service Uptake OR (95% CI)	*p*-value	Immediate Newborn Care Practices OR (95% CI)	*p*-value
Caregiver Sex	Male	Ref		Ref		Ref		Ref	
	Female	1.28 (0.90–1.81)	0.169	2.43 (1.66–3.53)	< 0.001	0.83 (0.56–1.23)	0.357	2.79 (1.97–3.95)	< 0.001
Marital status	Unmarried	Ref		Ref		Ref		Ref	
	Married	2.15 (1.33–3.47)	0.002	2.68 (1.72–4.18)	< 0.001	3.18 (1.39–7.28)	0.006	1.98 (1.40–2.81)	< 0.001
Household size (standardized)	Continuous	1.01 (0.88–1.14)	0.924	0.82 (0.71–0.94)	0.005	1.05 (0.90–1.23)	0.520	0.78 (0.69–0.89)	< 0.001
Survey year	2021	Ref		Ref		Ref		Ref	
	2022	0.94 (0.68–1.31)	0.720	1.11 (0.67–1.84)	0.695	0.73 (0.50–1.07)	0.102	0.88 (0.68–1.13)	0.310
	2023	1.34 (0.95–1.88)	0.091	2.64 (1.88–3.72)	< 0.001	0.96 (0.66–1.41)	0.836	0.92 (0.72–1.18)	0.510
	2024	0.86 (0.63–1.18)	0.357	2.10 (1.48–2.99)	< 0.001	0.89 (0.61–1.29)	0.525	0.71 (0.56–0.90)	0.004

At multivariate analysis, for adequate dietary diversity, the adjusted model demonstrated several statistically significant associations. Female caregivers remained more likely to achieve minimum dietary diversity compared with male caregivers (aOR≈2.1, *p* < 0.01). Married caregivers also maintained significantly higher odds of adequate dietary diversity (aOR≈2.8, *p* < 0.01). Household size remained inversely associated with dietary diversity (aOR≈0.75, *p* < 0.01), suggesting that larger households face greater challenges in maintaining diverse diets. Literacy status showed a modest positive association with dietary diversity after adjustment. Differences across survey years were attenuated after controlling for household and caregiver characteristics, suggesting that the observed temporal variation was partly explained by changes in household composition and caregiver characteristics.

For immediate newborn care practices, the adjusted model also produced stable estimates. Female caregivers had higher adjusted odds of practicing appropriate newborn care (aOR≈3.4, *p* < 0.001). Married caregivers remained more likely to report appropriate newborn care practices (aOR≈1.9, *p* < 0.05). Household size continued to show a negative association (aOR≈0.72, *p* < 0.01). Some caregiver occupation and displacement categories showed modestly lower odds of appropriate newborn care practices, though these effects were smaller in magnitude.

For appropriate IYCF practices and MNH service uptake, adjusted models showed limited additional explanatory power beyond marital status. After controlling for other covariates, most predictors were not significantly associated with the outcomes, suggesting that caregiver marital status and broader contextual factors may play a stronger role in influencing these practices. Model diagnostics indicated no evidence of model instability or separation after collapsing sparse categories and standardizing predictors as represented in Table 6.

**Table 6:** Multivariate regression analysis with adjusted odds ratio for outcomes.

Predictor	Category	Adequate dietary diversity aOR (95% CI)	*p*-value	Immediate newborn care practices aOR (95% CI)	*p*-value
Caregiver sex	Male	Ref		Ref	
	Female	2.22 (1.37–3.58)	0.001	3.41 (2.10–5.53)	< 0.001
Caregiver literacy	Cannot read/write	Ref		Ref	
	Literate	1.46 (1.05–2.04)	0.024	1.32 (1.02–1.70)	0.035
Marital status	Unmarried	Ref		Ref	
	Married	3.07 (1.65–5.73)	< 0.001	1.92 (1.17–3.14)	0.010
Household size (standardized)	Continuous	0.75 (0.61–0.92)	0.006	0.72 (0.60–0.87)	< 0.001
Occupation	Unemployed	Ref		Ref	
	Farmer/Laborer	0.89 (0.61–1.29)	0.540	0.81 (0.60–1.10)	0.180
	Small business	1.12 (0.74–1.69)	0.600	0.94 (0.69–1.29)	0.720
Displacement status	Host community	Ref		Ref	
	Internally displaced	0.76 (0.54–1.08)	0.130	0.83 (0.62–1.12)	0.220
	Refugee	0.68 (0.48–0.97)	0.034	0.79 (0.58–1.07)	0.130
Survey year	2021	Ref		Ref	
	2022	1.02 (0.74–1.40)	0.920	0.93 (0.72–1.20)	0.570
	2023	1.18 (0.85–1.64)	0.320	0.91 (0.70–1.18)	0.470
	2024	1.09 (0.79–1.51)	0.600	0.73 (0.56–0.95)	0.021

## Discussion

This study aimed to examine maternal and child health service use, health-seeking behaviors, and nutrition patterns and practices in three Sudan states in 2021 through 2024. Generally, the results suggest a pattern widely reported in fragile and crisis-affected contexts: initial engagement with services may remain relatively high, but continuity of care, repeated contacts, and time-sensitive maternal and newborn interventions are more vulnerable to disruption (19, 20). This pattern can be interpreted through several complementary frameworks. The Three Delays model helps explain why home delivery may remain common despite ANC attendance, particularly where insecurity, transport barriers, and service access constraints affect care-seeking and timely referral (19). The WHO continuum-of-care framework similarly emphasizes that maternal and newborn health gains depend not merely on first contact with care, but on sustained, quality interactions across pregnancy, childbirth, and the postnatal period (21, 22). In addition, the UNICEF conceptual framework highlights how child feeding outcomes are jointly shaped by caregiving resources, food access, service availability, and broader household constraints (20). The socio-demographic gradients observed in this study are also consistent with Andersen's behavioral model, under which predisposing and enabling factors influence service utilization and care practices (23, 24).

Home delivery remained the dominant pattern across the study period despite relatively high levels of any ANC attendance. This finding suggests that entry into maternal health care does not necessarily translate into continuity of care or facility-based childbirth. Similar patterns have been documented in fragile and conflict-affected contexts, where insecurity, transport barriers, opportunity costs, poor perceived quality of care, and disrupted referral systems reduce women's ability to complete the maternal care continuum (25, 26). A systematic review of antenatal care utilization in fragile and conflict-affected situations found that the most frequently reported determinants of ANC use included education, socioeconomic status, perceived quality of care, and access-related barriers, while conflict often intensified rather than replaced these underlying constraints (26). Evidence among Syrian refugees in Jordan and Lebanon similarly showed that maternal health service utilization was molded not only by humanitarian support, but also by service costs, health system design, and availability of care (25). These contrasts suggest that the relatively high prevalence of at least one ANC visit in the present study may mask continuity of care weaknesses.

The deterioration in repeated ANC attendance is especially imperative in light of WHO recommendations for a minimum of eight antenatal contacts for a positive pregnancy experience (21). The WHO ANC model was introduced in acknowledgment of the fact that timely recurrent contacts improve opportunities for clinical monitoring, counseling, birth preparedness, and timely identification of complications (21). In this study, reduced higher-order ANC attendance indicates that women may be entering care but not sustaining contact across pregnancy. Evidence from low-resource settings outside active war zones, including eastern Uganda, has similarly shown that attaining the recommended 8+ ANC contacts remains difficult, suggesting that continuity barriers may be even more definite in humanitarian contexts (27). The present findings therefore point to a contact-continuity gap rather than a complete absence of service engagement.

Postnatal care and immediate newborn care appeared to be among the most unstable components of the maternal-newborn continuum. This is replicable with broader literature, which indicates that the immediate postnatal period is one of the most vulnerable stages for service disruption because it requires timely contact, skilled attendance, and quality-sensitive practices soon after birth (22, 28). WHO recommendations identify the first days after delivery as a critical window for both maternal and neonatal survival and stress the importance of routine postnatal contacts and essential newborn care actions (22). A systematic review of maternal and newborn intervention delivery in conflict settings likewise found that most documented interventions focused more profoundly on antenatal and obstetric care, with far less programmatic emphasis on postnatal and newborn care delivery, especially through community-level platforms (28). This may help explain why postnatal attendance and immediate newborn care practices in the present study were more volatile than ANC use.

At the same time, caution is needed in interpreting year-to-year fluctuations in specific newborn care indicators. Some practices may have changed substantially due to real improvements or deterioration in care quality, but abrupt shifts may also reflect differences in questionnaire wording, probing, coding, recall, or changes in the proportion of home versus facility births captured across survey rounds. This apprehension is reinforced by WHO/UNICEF IYCF measurement guidance, which stresses the importance of standardized definitions and consistent measurement when comparing indicators over time (29). Accordingly, some of the more dramatic annual changes in immediate newborn care practices should be treated as signals requiring verification rather than definitive proof of behavioral change.

The deterioration in breastfeeding indicators and the reported increase in pre-lacteal feeding are also consistent with the literature from conflict and emergency settings. A systematic review of Maternal, infant, and young child nutrition practices in conflict areas found that breastfeeding is frequently undermined by displacement, maternal stress, mental health challenges, disruption of family support systems, poor access to counseling, and inappropriate availability or circulation of breastmilk substitutes (30). In humanitarian contexts, mothers may adopt pre-lacteal feeding or early supplementation as coping strategies in response to stress, perceptions of insufficient milk, or disrupted support, even though such practices increase the risk of poor breastfeeding outcomes and child morbidity (30, 31). The findings in this study therefore align with evidence that suboptimal breastfeeding in emergencies is not simply a matter of caregiver choice, but is shaped by broader social, psychological, and system-level pressures.

This understanding is further strengthened by international guidance on IYCF in emergencies. WHO and UNICEF emphasize early initiation of breastfeeding, avoidance of pre-lacteal feeding, continued breastfeeding, and strong standardization of IYCF measurement (29). The Operational Guidance on Infant and Young Child Feeding in Emergencies also stresses the need for protected breastfeeding support, careful management of breastmilk substitutes, and skilled counseling in crisis settings (31). The breastfeeding patterns observed in this study therefore suggest a need to view infant feeding as a core protection and health systems issue within humanitarian programming.

Dietary diversity showed marked interannual fluctuation, improving sharply and then declining again, which suggests that child diet quality in crisis settings is highly sensitive to changing contextual conditions. Under the UNICEF conceptual framework, child diets are immediate determinants of nutritional status, but they are in turn shaped by underlying household food access, caregiving capacity, health services, and the broader social and economic environment (20). This framework corresponds closely with the present findings, in which displacement status and household size remained important predictors of adequate dietary diversity after adjustment.

The lower odds of adequate dietary diversity among refugee households are plausible and consistent with broader humanitarian evidence. Forced displacement often reduces access to livelihoods, stable food markets, purchasing power, and social support, while increasing reliance on assistance mechanisms that may protect total food access more readily than food variety (20, 32, 33). Larger households may also face resource dilution, whereby limited food, time, and caregiving attention are spread more thinly across members. Evidence from systematic reviews and meta-analyses of cash transfer programmes indicates that cash support targeted to households with young children can improve child nutritional outcomes, partly through pathways involving improved dietary diversity and increased consumption of nutrient-rich foods (32, 33). These findings support the interpretation that dietary diversity in this study was likely influenced by both structural displacement-related disadvantage and household-level resource constraints.

The regression findings showed a clear socio-demographic gradient across outcomes. Female caregivers, literate caregivers, and married caregivers had higher odds of favorable nutrition and newborn care outcomes, while larger household size was negatively associated with both. These patterns are consistent with the literature on maternal and child health service use in fragile settings, where education, household support, and socioeconomic position often shape care-seeking behavior, caregiving knowledge, and capacity to act on health information (23, 24, 26). In the context of Andersen's behavioral model, literacy and marital status can be understood as enabling factors that improve access to knowledge, support, and practical decision-making resources, whereas larger household size may constrain both caregiving time and resource allocation (23, 24).

The adjusted analyses also help clarify the meaning of temporal trends. Some apparent year effects weakened after controlling for caregiver and household characteristics, suggesting that part of the observed variation across rounds may reflect differences in population composition rather than a simple linear programme effect. This is especially important in repeated cross-sectional studies conducted in dynamic humanitarian settings, where changes in displacement mix, household size, and caregiver profile may alter observed prevalence over time. However, when negative year effects remain after adjustment, particularly for quality-sensitive newborn care practices, they may point more strongly to a genuine deterioration in service or care conditions, although causal interpretation should still be avoided (23, 24).

Essentially, the findings suggest that maternal, newborn, and nutrition programming in humanitarian settings should focus not only on initial service contact, but also on continuity, quality, and the household conditions that enable appropriate care and feeding practices. First, the gap between any ANC and repeated ANC contacts indicates a need for stronger continuity-support strategies, including outreach services, predictable scheduling, reduced waiting times, and follow-up systems for missed visits, in line with WHO's antenatal care recommendations (21). Second, the instability in postnatal care and newborn care practices highlights the need to protect the immediate postnatal period through both facility-based quality improvement and community-linked follow-up systems, consistent with WHO postnatal care guidance (22, 28). Third, the deterioration in breastfeeding indicators supports galvanization of IYCF-E interventions into ANC, childbirth, PNC, and community services, including skilled counseling, peer support, and stricter management of breastmilk substitutes (29, 31). Lastly, dietary diversity findings suggest that market-sensitive “cash-plus” approaches combining transfer support with nutrition counseling may be particularly vital for displaced households and larger families (32, 33).

## Recommendations

To sustainably improve maternal and child nutrition outcomes in Sudan, future interventions should prioritize integrated, community-based approaches that strengthen both knowledge and access. This includes enhancing community-level nutrition education with a strong focus on optimal infant and young child feeding practices, especially in emergencies, while protecting and promoting breastfeeding through practical measures such as establishing mother-baby safe spaces in displacement settings and training frontline health workers to provide breastfeeding support in crisis contexts. Improving dietary diversity should also be a key priority, through the integration of food security and nutrition-sensitive agricultural initiatives that increase the availability and affordability of diverse, nutritious foods. In parallel, culturally sensitive behavior change communication strategies are needed to address harmful feeding practices, improve uptake of health services, and rebuild community trust in health systems.

To support the delivery of essential health services, interventions should focus on expanding community-based outreach through mobile clinics and trained village midwives to improve coverage in remote and conflict-affected areas, while also restoring and rehabilitating damaged health facilities to ensure adequate supplies, staffing, and operational readiness. Emergency referral systems must be strengthened through reliable transport and communication channels to enable timely care for maternal and child health emergencies. Outreach services should adopt an integrated model that combines maternal, newborn, child health, and nutrition services with routine immunization and health education. At the same time, nurturing community engagement through participatory approaches can improve health literacy, strengthen local accountability, and increase service acceptance and utilization. These efforts must be executed with an explicit equity focus to ensure that displaced, rural, and otherwise underserved populations are adequately reached through inclusive planning and resource allocation. These multi-pronged, community-driven strategies are essential not only for meeting immediate humanitarian needs but also for building long-term resilience within Sudan's fragile health system.

### Strengths and Limitations

Key strengths of this study include its extensive temporal coverage from 2021 to 2024, capturing evolving conditions and responses, as well as its geographic diversity covering multiple regions with varying degrees of conflict and service availability. This broad coverage enhances the reliability and applicability of the identified trends and facilitates targeted recommendations for policymakers and humanitarian actors. The study's limitations include reliance on caregiver recall and self-reported practices, which may introduce recall and social desirability biases. The timing of the study presents a critical and vulnerable period in Sudan, and doesn't necessarily reflect an ideal status quo, and the study's results are limited to similar settings. Additionally, the absence of direct measurements of nutritional status restricts precise interpretations regarding malnutrition prevalence and severity. Some immediate newborn care indicators were measured only in later rounds, limiting the strength of trend interpretation. The repeated cross-sectional design also does not permit causal inference, and residual confounding is likely. Self-reported practices are subject to recall and social desirability bias, while possible differences in survey implementation across rounds may affect comparability (27).

Nonetheless, the study contributes important evidence by examining multiple years of maternal, newborn, and IYCF indicators together and situating those results within both household-level predictors and the wider literature from fragile and humanitarian settings. Despite these constraints, the consistency of observed trends with broader national data supports the validity and relevance of the study findings. Furthermore, only verbal consent was taken. A written informed consent with details of survey confidentiality, and voluntary participation would have strengthened the ethical rigor and documentation of the study. However, a decision was taken for these to be read out to the study participants.

## Data Availability

The original contributions presented in the study are included in the article/Supplementary material, further inquiries can be directed to the corresponding author.
